# Supplementary material for: From sequence to enzyme mechanism using multi-label machine learning
Source: BMC Bioinformatics. 2014 May 19;15:150. doi: 10.1186/1471-2105-15-150 (PMC4229970; doi:10.1186/1471-2105-15-150)
Supplement: Additional file 2 — Java code of ml2db. Additional file ml2db_code.tar.gz contains the Java source code to run the multi-label machine learning experiments and save the results to database. The code’s Javadoc is included. [file 1471-2105-15-150-S2.zip › additional file 2/ml2db/ml2db/doc/mulan/classifier/neural/model/class-use/Neuron.html]

Uses of Class mulan.classifier.neural.model.Neuron


JavaScript is disabled on your browser.


- Overview
- Package
- Class
- Use
- Tree
- Deprecated
- Index
- Help

- Prev
- Next

- Frames
- No Frames

- All Classes

## Uses of Class mulan.classifier.neural.model.Neuron

- Packages that use Neuron

  | Package | Description |
  | --- | --- |
  |  |  |
  | --- | --- |
  | mulan.classifier.neural |  |
  | mulan.classifier.neural.model |  |
- - ### Uses of Neuron in mulan.classifier.neural

    Constructor parameters in mulan.classifier.neural with type arguments of type Neuron

    Constructor and Description

    `MMPMaxUpdateRule(java.util.List<Neuron> perceptrons,
    RankingLossFunction lossMeasure)`

    Creates a new instance of `MMPMaxUpdateRule`.

    `MMPRandomizedUpdateRule(java.util.List<Neuron> perceptrons,
    RankingLossFunction lossMeasure)`

    Creates a new instance of `MMPRandomizedUpdateRule`.

    `MMPUniformUpdateRule(java.util.List<Neuron> perceptrons,
    RankingLossFunction lossMeasure)`

    Creates a new instance of `MMPUniformUpdateRule`.

    `MMPUpdateRuleBase(java.util.List<Neuron> perceptrons,
    RankingLossFunction loss)`

    Creates a new instance of `MMPUpdateRuleBase`.
  - ### Uses of Neuron in mulan.classifier.neural.model

    Methods in mulan.classifier.neural.model that return types with arguments of type Neuron

    | Modifier and Type | Method and Description |
    | --- | --- |
    |  |  |
    | --- | --- |
    | `java.util.List<Neuron>` | NeuralNet.`getLayerUnits(int layerIndex)` Returns units of a particular layer of the neural network. |
    | `java.util.List<Neuron>` | BasicNeuralNet.`getLayerUnits(int layerIndex)` |

    Methods in mulan.classifier.neural.model with parameters of type Neuron

    | Modifier and Type | Method and Description |
    | --- | --- |
    |  |  |
    | --- | --- |
    | `boolean` | Neuron.`addNeuron(Neuron neuron)` Adds a connection to a specified `Neuron`. |
    | `boolean` | Neuron.`removeNeuron(Neuron neuron)` Removes a connection to a specified `Neuron`.  The passed instance is assumed to be connected to the output of this instance (forward connections only). |

    Method parameters in mulan.classifier.neural.model with type arguments of type Neuron

    | Modifier and Type | Method and Description |
    | --- | --- |
    |  |  |
    | --- | --- |
    | `boolean` | Neuron.`addAllNeurons(java.util.Collection<Neuron> neurons)` Adds connections to all specified `Neuron` instances.  Each instance of the collection is assumed to be connected to the output of this instance (forward connections only). |

    Constructor parameters in mulan.classifier.neural.model with type arguments of type Neuron

    Constructor and Description

    `Neuron(ActivationFunction function,
    int inputDim,
    double biasValue,
    java.util.Collection<Neuron> nextNeurons)`

    Creates a new `Neuron` instance.

- Overview
- Package
- Class
- Use
- Tree
- Deprecated
- Index
- Help

- Prev
- Next

- Frames
- No Frames

- All Classes
